# Patterns of Sedentary Behavior in a Community-Based Cohort of Older Adults

**DOI:** 10.1093/geroni/igaa057.2862

**Published:** 2020-12-16

**Authors:** Dori Rosenberg, Rod Walker, Mikael Anne Greenwood-Hickman, KatieRose Richmire, John Bellettiere, David Wing, Andrea LaCroix

**Affiliations:** 1 Kaiser Permanente Washington, Seattle, Washington, United States; 2 Kaiser Permanente Washington Health Research Institute, Seattle, Washington, United States; 3 University of California San Diego, La Jolla, California, United States

## Abstract

Few epidemiologic studies have examined device-measured sitting patterns by demographics and health status. The Adult Changes in Thought (ACT) study is an on-going epidemiologic study of adults age ≥65 years. We conducted a sub-study that added a thigh-worn activPAL device and sleep logs for 7 days to the measurement protocol. A total of 997 had valid wear time (≥4 days with 10-20 hours of data per day) and covariate data. activPAL sedentary pattern measures included number of sitting bouts lasting 30 minutes or more and mean sitting bout duration. On average, participants (56% female, 57% > age 75, 89% non-Hispanic white) sat in bouts lasting 17 minutes (SD = 12) and had 5.9 (SD = 1.7) bouts of sitting lasting 30 minutes or more. Participants who were older, were male, had obesity, had worse self-rated health, had depression, and had difficulty walking had longer sitting bouts and more prolonged bouts.

